# 3D bioprinting for cancer modeling and drug screening

**DOI:** 10.1186/s40364-026-00951-x

**Published:** 2026-06-20

**Authors:** Aurélie Cadiou, Eva-Laure Peiller Matera, Nicolas Ellinger, Lars Petter Jordheim, Michael Duruisseaux, Christophe Marquette, Charles Dumontet

**Affiliations:** 1 Cancer Research Center of Lyon, UMR INSERM 1052, CNRS 5286, Université Claude Bernard Lyon 1, Lyon, France; 2 Antineo, Lyon, France; 3 Laboratoire de Biologie Tissulaire et d’Ingénierie Thérapeutique, Université Claude Bernard Lyon 1, CNRS UMR 5305, IBCP, Lyon, France; 4 Hospices Civils de Lyon, Lyon, France; 5 3d.FAB, Université Claude Bernard Lyon 1, CNRS, INSA, CPE-Lyon, ICBMS, UMR 5246, Villeurbanne, France

**Keywords:** Preclinical models, 3D bioprinting, Drug screening, Primary samples

## Abstract

3D bioprinting has emerged as a promising preclinical technology to explore tumor cell biology, interaction with the microenvironment, and drug sensitivity. Current methods allow inclusion of several cell types within the bioprinted objects and may also be compatible with migration of immune cells within the object. 3D bioprinted tumor models have been shown to be more relevant for preclinical studies than classical 2D systems for a number of key parameters including cell morphology, cell-cell interactions, interaction with non-tumor cells, drug diffusion and sensitivity. As compared to other 3D models, bioprinting is applicable to a large variety of tumor cell lines at a relatively low cost and may be performed over a flexible period of time, ranging from a few days to several weeks. Bioprinting has also increasingly been applied to primary samples from cancer patients. In this review, we present the promises and technical hurdles yet to overcome in order for 3D bioprinting models to be fully exploited in preclinical modeling of cancer.

**Supplementary Information:**

The online version contains supplementary material available at https://doi.org/10.1186/s40364-026-00951-x.

## Introduction

Drug discovery and development is an expensive [1], high-risk, and long process lasting 10 to 15 years [2, 3]. Unfortunately, there are high attrition rates at every stage due to lack of efficacy and/or safety issues [4], and only a fraction of candidates selected by the preclinical phase actually succeed in clinical trials [2, 5, 6]. One study reported that only 31.8% of drug candidates advance from preclinical development to Phase I clinical trials, and estimated the likelihood of approval (LOA) from Phase I to be 19.3% across all indications [7]. Another study estimated the LOA from Phase I to be 13.8% across all indications and showed significant variations by therapeutic area, ranging from a lowest value of 3.4% in oncology to a maximal value of 33.4% for vaccines (infectious diseases) [8].

In the preclinical phase, drug candidates are tested in several in vitro and in vivo models to select the most effective and safe compounds for human clinical trials. The very high attrition rate between preclinical and early phase clinical trials is likely to result from a number of technical limitations, including an insufficient correlation between preclinical anticancer activity assays and responses observed in patients, as well as the lack of reliable predictive animal models of toxicity. The poor correlation between preclinical in vitro and in vivo data with clinical trial results is well documented [3] and is unfortunately compounded by the dearth of disease-relevant preclinical models [9]. Key limitations include the inability to replicate the human tumor microenvironment (TME), capture tumor heterogeneity, and model the mechanisms underlying drug resistance.

For the past decades, to understand the underlying cellular mechanisms that lead to human disease and to test new therapeutic compounds, scientists have mainly relied on cells seeded in two-dimensional (2D) surfaces and animal models. Although classical cell culture systems have provided valuable insights into the fundamentals of cell biology, they are too simplistic to accurately replicate the complexity of human tumors, where cell-cell and cell-extracellular matrix (ECM) interactions play critical roles. Cells cultured in 2D show irrelevant cell proliferation and differentiation [10, 11], gene expression [12, 13], and sensitivity to treatments [11, 14, 15]. Animal models provide a more physiologically relevant representation of the TME than 2D in vitro cultures [16]. However, xenograft models based on the subcutaneous implantation of human tumor cells into immunocompromised animals often poorly predict therapeutic responses in human patients. This limitation can be partially overcome by using patient-derived xenograft (PDX) models in humanized mice [17, 18]. Despite these advances, their translational relevance remains constrained by the persistence of rodent-derived stroma and the lack of a fully functional human immune system, which hinders the assessment of immunotherapy [19]. Furthermore, these models are heterogeneous, time-consuming, costly, and raise ethical concerns.

Physiologically relevant in vitro models that better emulate human biology and disease are urgently needed to improve drug development pipelines and advance personalized medicine. In this context, 3D cell models are emerging to bridge the gap between 2D systems and animal models (Table 1), increasing the efficiency and accuracy of preclinical workflows. While 3D models are not likely to completely replace animal experimentation, they can increase the predictive value of drug screening, and reduce the number of animals used, in alignment with the 3Rs principles (Replace, Reduce, and Refine). 3D tumor models present multiple advantages. First, they allow complex cell-cell and cell-matrix interactions, preserving the physiological cellular phenotype [12, 20–22]. These models also aim to replicate the natural tumor architecture by exhibiting three areas: (i) an exterior rim containing highly proliferative cells, (ii) an intermediate hypoxic zone with a low oxygen concentration and nutrients, and (iii) an anoxic core enclosing necrotic cells [23, 24]. The differences in oxygen, nutrient, and pH gradients trigger hypoxia- and stress-related signaling pathways associated with treatment resistance, making 3D models highly relevant models for investigating drug penetration and therapeutic efficacy. As a result of this increased complexity and biomimicry, gene expression patterns resemble those of the tumor tissue in a patient, leading to a more relevant response to treatment and enhanced predictive value [25, 26] underlining their great potential as a preclinical tool.

**Table 1:**
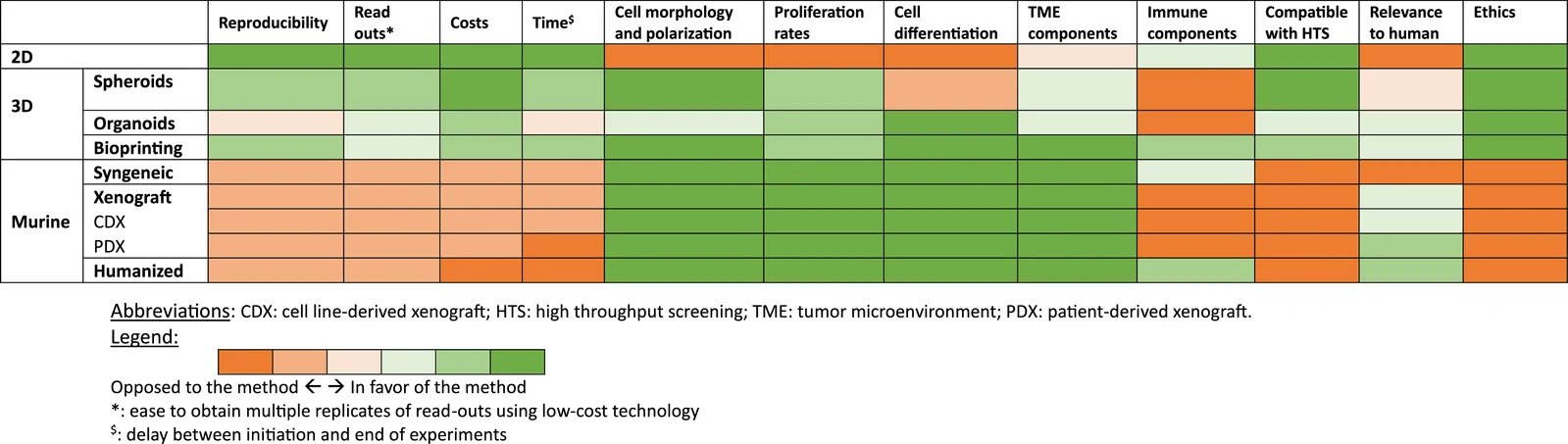
Relative advantages and limitations of 2D/3D/murine models

**Fig. 1:**
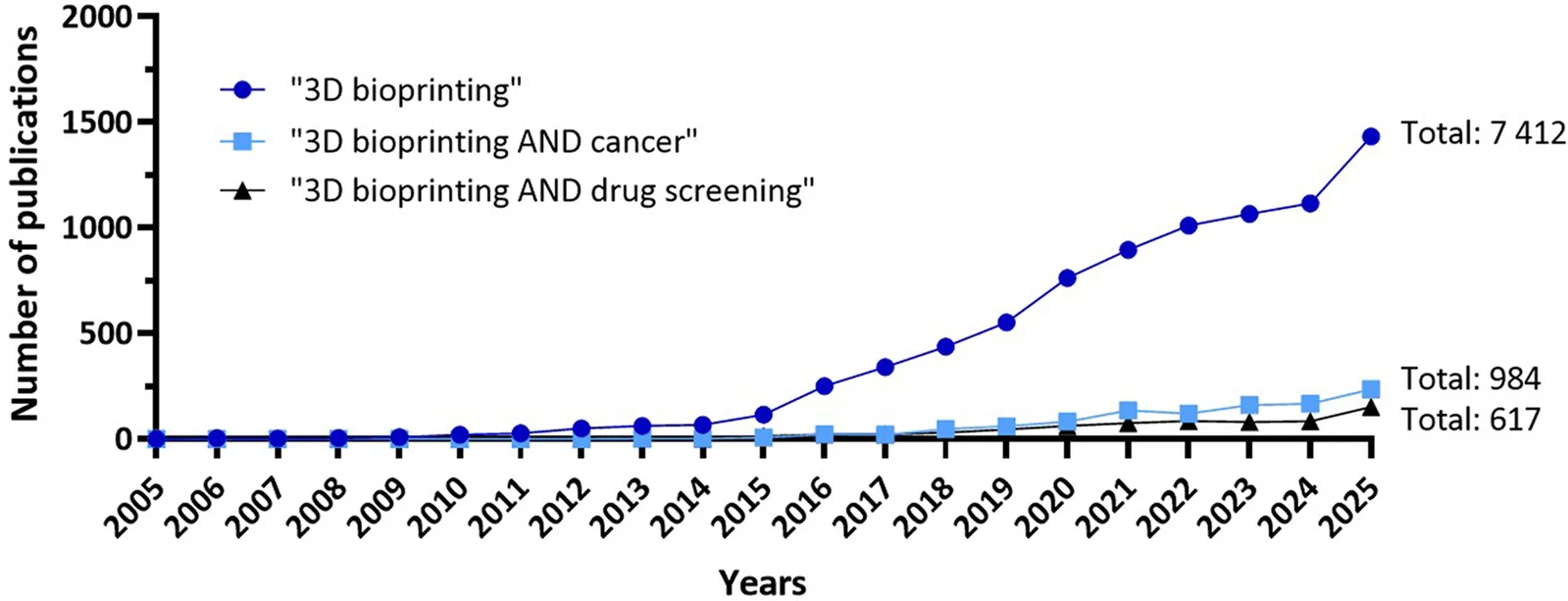
Evolution of the number of published papers in PubMed database, using the keywords “3D bioprinting”, “3D bioprinting AND cancer” or “3D bioprinting AND drug screening” from 2005 to 2025

Multiple engineering approaches exist, allowing the modeling of cancer in 3D. The main 3D models currently used include spheroids, organoids, and 3D bioprinted models. Spheroids are defined as free-floating cell clusters with or without scaffold [27–29] and organoids as multicellular in vitro structures derived from cells that have the capacity to self-organize and differentiate to form a tissue-resembling object [30, 31]. Although spheroids and organoids are valuable models, they are limited in terms of control over the spatial organization of diverse cell types in complex architectures, and their production processes often lacks consistency and reproducibility [32]. Spheroids are used to mimic 3D interactions and potentially reduce drug diffusion within a cellular aggregate but do not exhibit tissue-specific architecture or organized cellular differentiation. In contrast, organoids, or their neoplastic counterparts tumoroids, are able to preserve tumor heterogeneity and architectural organization, offering enhanced physiological relevance relative to standard 2D cultures and simple spheroid systems. However, their dependence on ECM scaffolds (e.g., Matrigel™) and specific culture media formulations results in high experimental variability and increased experimental complexity [33]. Recently, 3D bioprinting has been increasingly reported (Fig. 1) as a promising new approach for producing complex 3D biological constructs for disease modeling [34] and drug screening, particularly in cancer research [35–37]. However, references specifically dealing with drug screening in oncology are scarcer, with a maximum value of 69 references in 2025. This technique addresses the limitations of traditional 3D model development by enabling the precise placement of multiple cell types and biomaterials to replicate the tumor microenvironment, while offering full control over the model’s size, shape, matrix composition, and stiffness to reproduce the architecture of the studied cancer type. Interestingly, bioprinted cancer models, which typically measure around a hundred cubic micrometers but may also reach larger volumes, are more likely to mimic the actual diffusion barriers encountered by drugs in vivo. In this review, we focus on 3D bioprinting technology and its use for cancer modeling and drug screening.

## 3D bioprinting

3D bioprinting is an additive manufacturing technology utilizing cell-containing biomaterials, known as bioinks [34], to construct 3D in vitro models in a layer-by-layer manner [35, 36]. This process enables the precise positioning of different cell types in a spatially controlled and reproducible manner, allowing for the creation of complex 3D architectures [37]. The matrix composition and rigidity can be adapted either locally or globally according to the cancer model being studied. The rapid automated fabrication of models can be used to generate biomimetic constructs for high-throughput in vitro drug screening. Detailed description of the 3D bioprinting process have been described elsewhere [38–41] and summarized in the Supplementary Fig. 1.

The choice of the bioink components is of particular importance in cancer models since the composition may have an impact on tumor cell interaction and signaling with its environment, tumor cell migration, drug diffusion or ability of immune cells to migrate within the bioprinted object. Although most bioinks contain isolated cells, they can also incorporate pre-formed cellular aggregates, such as spheroids [42–49] or organoids [50]. These bioinks offer several advantages, including enhanced cell-cell interactions, improved biomimicry, accelerated tissue formation, and long-term maintenance of cell phenotypes [51]. Nevertheless, their use presents notable challenges, such as the large cell number required to produce sufficient quantities of aggregates, and the limited permeability of the aggregates when the size exceeds 400 μm in diameter, compromising cell viability [52]. The requirements needed for 3D bioprinting cannot be met by a single polymer, which is why researchers have developed multicomponent matrix. These matrix components enable the enhancement of functionality and complexity of printed objects [53]. The polymers used can either be of natural, synthetic, or semi-synthetic origin (Supplementary Table 1 and Supplementary Fig. 2), and they are usually used as hydrogels [54]. Since extrusion technology is the most widely used bioprinting method, many of the biomaterials are used in this context. However, these biomaterials are not limited to extrusion technology and could be used with other technologies mentioned below.

Natural polymers, such as alginate, collagen, gelatin, and hyaluronic acid, are commonly used due to their inherent biocompatibility and ability to polymerize under mild conditions [22]. Furthermore, gelatin and collagen are widely used because they contain structural motifs (e.g., protein ligands) that are present in the ECM, which can enhance extracellular signaling and support cell proliferation, differentiation, and function [55, 56]. This contrasts with alginate and hyaluronic acid, which are polysaccharides that lack inherent protein ligands. The latter typically require chemical functionalization, such as grafting arginine–glycine–aspartic acid (RGD) peptides [57], or use in conjunction with another biomaterial, to enable active cell adhesion and signaling [58–60]. For instance, Wang et al. combined type I collagen with hyaluronic acid. The collagen provided a scaffold for cell adhesion, proliferation, and tissue-like structure formation, while the hyaluronic acid enhanced the hydrogel’s viscosity, water retention, and biocompatibility [61]. Growth factors can also be entrapped within scaffolds to direct cell differentiation, and be released passively as the network degrades [55]. To better recapitulate the ECM glycosylation, Cadamuro et al. developed a method to generate glycosylated 3D tissue models [62]. The main limitations are the weak mechanical properties and the high batch-to-batch heterogeneity of natural polymers derived from mammalian ECM, lowering reproducibility between experiments [63].

In contrast, the properties of synthetic biomaterials, such as their mechanical strength, degradation rate, and bioactivity, can be easily tuned [55, 56]. To promote cell-matrix interactions, bioactive compounds must be added to or grafted onto the network, such as peptide sequences. The most commonly used peptide is the three-amino-acid fibronectin-derived peptide RGD [64], which binds to αvβ3 and αvβ5 integrins [33]. Synthetic polymers include pluronics, polyethylene glycol (PEG), polylactic acid (PLA), and polycaprolactone (PCL). However, they are seldom used because of the stringent polymerization conditions needed and their physiological irrelevance.

Semi-synthetic polymers, that combine the biocompatibility of natural polymers with the beneficial characteristics of synthetic materials, are widely employed. Commonly semi-synthetic polymers used are gelatin methacrylate (GelMA), hyaluronic acid methacrylate (HAMA) and methylcellulose.

Replicating the complexity of the native ECM of the tumor or its normal tissue counterpart using these polymers or a mixture of them remains a significant challenge, as each tissue has a unique ECM composition [65]. Decellularized ECM (dECM), which retain the structural proteins, glycosaminoglycans, glycoproteins, and bioactive signals of the native matrix, offer a promising alternative [66, 67]. They can be made from different tissues such as porcine breast [68–71] or liver tissue [72, 73]. However, dECM typically requires combination with other biomaterials to overcome its poor mechanical properties [71], which alters its native composition and impairs faithful tissue reconstruction [74]. Matrigel™ has been used as a means to replicate the tumor stroma [44, 75–78]. Recently, Shukla et al. developed a caprine-derived dECM bioink compatible with extrusion-based bioprinting that does not require additional viscosity modifiers [79].

**Fig. 2:**
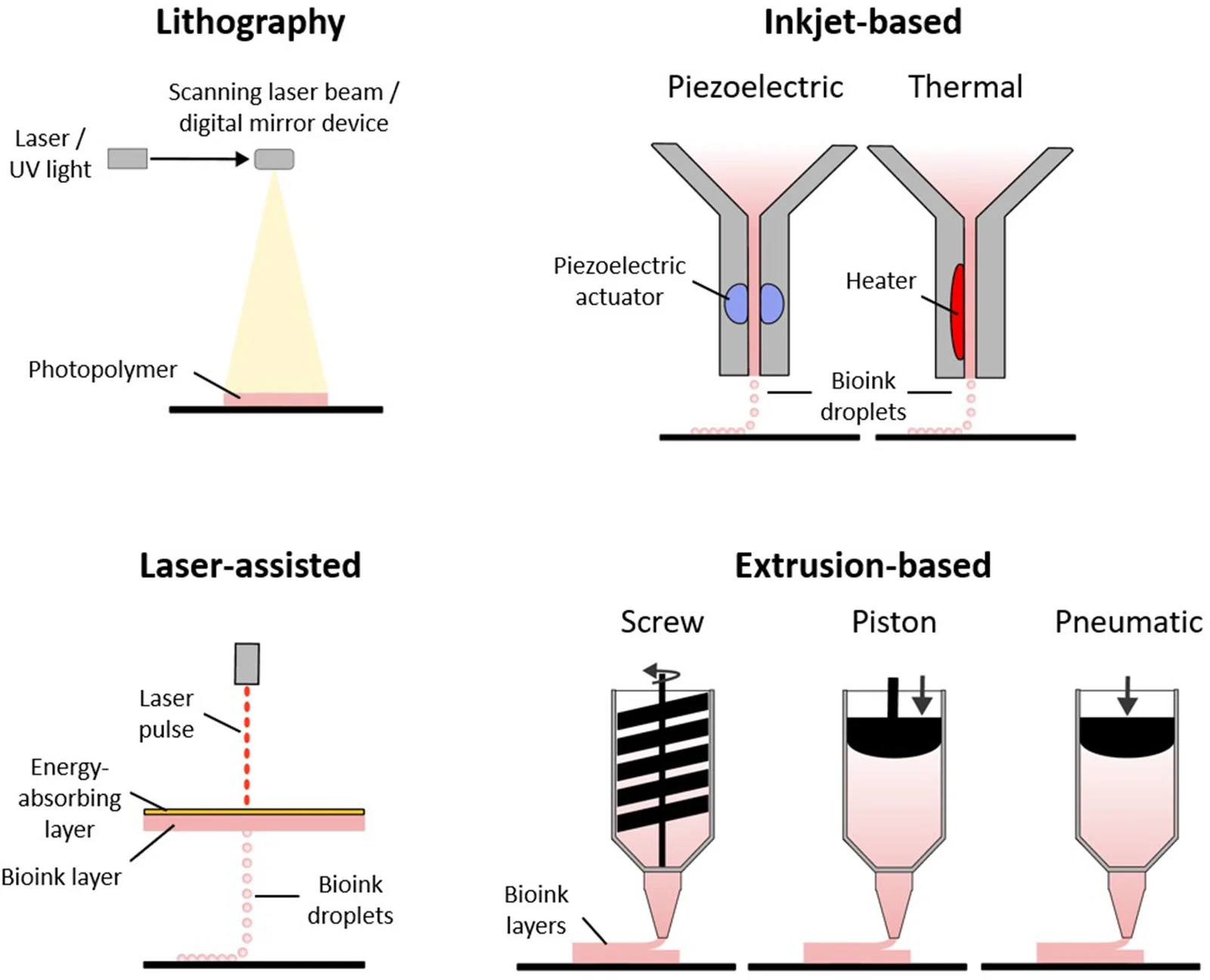
Schematic illustrations of different bioprinting techniques: lithography, inkjet-based, laser assisted and extrusion-based bioprinting

The type of polymer chosen, the desired rigidity, and the expected resolution will determine the bioprinting method to be used. Although a wide range of bioprinting techniques are available, four main classes are most commonly described: (i) lithography, (ii) inkjet-based, (iii) laser-assisted (LAB), and (iv) extrusion-based bioprinting (EBB) [39, 80] (Fig. 2). Each of these methods has its advantages and drawbacks in terms of resolution, speed, control, cost, and availability of compatible bioinks (Table 2).

**Table 2:** Comparison of the main parameters of bioprinting techniques

	Lithography	Inkjet-based	LAB = LIFT	EBB
Resolution	Very high (5–10 μm)	High (20–100 μm)	High (20–80 μm)	Low (> 100 μm)
Printing speed	High	Medium	Medium	Low-to-medium
Cell density	Medium (< 10^8^ cells/mL)	Low (< 10^6^ cells/mL)	Medium (< 10^8^ cells/mL)	High
Cell viability	High (> 85%)	High (> 85%)	High (> 90%)	Low-to-high (40–90%)
Bioinks	Photosensitive	Limited to low viscosity	Photosensitive	Wide range
Cost	High	Low	High	Medium

*Abbreviations*: EBB: extrusion-based bioprinting; LAB = LIFT: laser-assisted bioprinting = laser-induced forward transfer

### Lithography

Lithography-based technology uses light to photo-crosslink specific regions of cell-laden biomaterials layer by layer to form solid 3D constructs [39, 81]. Depending on how the light is delivered, lithography technologies can be divided into stereolithography (SLA), two-photon polymerization (2PP) [82], and DLP [38, 40]. A scanning laser beam is used in SLA and 2PP technologies, while a digital mirror device is used in DLP technology to rapidly mask light into 2D patterns [83]. These techniques have several advantages, including high spatial resolution, rapid printing speed of complex structures, and higher cell viability [84].

### Inkjet-based bioprinting

Inkjet-based bioprinting, utilizes thermal or electrical energy to eject droplets (1 to 100 picolitres) of biomaterials on a substrate to form 3D constructs [85]. In thermal inkjet bioprinting, a micro-heater is used to vaporize small volumes of the bioink, creating a pulse that ejects droplets from the nozzle. In piezoelectric inkjet bioprinting, a piezoelectric actuator directs a mechanical pulse to the bioink in the nozzle, generating a shock wave that propels it through [86]. This technique allows for high resolution [37] in a short amount of time and high cell viability after printing. However, one of the main limitations of inkjet technology is its inability to print materials with a high viscosity, such as natural extracellular matrix [86, 87].

### Laser-assisted bioprinting

LAB, also known as laser-induced forward transfer (LIFT), is a method that uses pulsed laser beam as an energy source to deposit cell encapsulating biomaterial droplets on a surface [38]. The energy created by the irradiation of the laser source is absorbed by an absorbent layer (made of gold or titanium), creating air bubbles on its surface and increasing the pressure. This process causes the absorbent layer to evaporate locally, generating high pressure and propelling the biomaterial onto a substrate in droplet form. Droplet size varies according to the biomaterial’s viscosity and the laser pulse energy [87]. The substrate can be composed of biopolymers or a culture medium [88]. This nozzle-free technology enables precise material deposition while preserving cell viability post printing without clogging issues [87]. Limitations of LAB are the high cost of the printer and the long fabrication time required.

### Extrusion-based bioprinting

EBB is one of the most widely used and studied techniques due to its affordability, ease of manipulation, and the ability to produce porous structures in a relatively short time, as in minutes [89]. EBB technique can also be found under the names 3D plotting, bioplotting, robotic dispensing, or microextrusion. By applying pneumatic pressure or mechanical force (piston or screw), the ink is pressed through a dispensing tip (e.g. a fine needle or a nozzle), allowing precise deposition of the biomaterials in the form of continuous filaments [38]. Piston-driven deposition gives more direct control over the ink than pneumatic systems because of the compressed gas delay in the latter system. Screw-based systems provide greater spatial control and are ideal for dispensing high-viscosity inks [86]. A wide range of inks is available with EBB technology, not only cell-loaded biomaterials, but also cell aggregates [90]. Printers are often equipped with multiple extruders, enabling users to print various bioinks in a single object [40]. The limitations of this technique are its low resolution (> 100 μm), and the high cell death during extrusion due to shear stress in the needle [90].

In order to maintain bioink structural integrity and stability after printing and for a period compatible with the biological experiments, the 3D structures must be crosslinked. This critical step also allows the stiffness of the extracellular environment to be adjusted according to the cancer model being studied. Different strategies exist depending on the biomaterials used, including physical (thermal or UV light), chemical (ionic or pH), and enzymatic crosslinking. Physical crosslinking using UV light, also known as photopolymerization or photo-crosslinking, requires that natural or synthetic polymers be modified with various functional groups, such as an acryloyl group (e.g., gelatin methacrylate), and the presence of a photo-initiator, such as lithium phenyl-2,4,6-trimethylbenzoylphosphinate (LAP). When exposed to UV radiations, photo-initiators form free radicals which initiate the photo-crosslinking reaction of photopolymerizable biomaterials [81]. However, these free radicals have been shown to induce cellular damage, particularly in sensitive primary cell samples [91]. While photopolymerizable bioinks are essential to lithography-based technologies, they can also be an option for extrusion-based bioprinting applications when the bioprinter is equipped with photocuring sources [92–94].

## 3D bioprinted cancer models

### Single cell type 3D bioprinted models

Bioprinting has been shown to be compatible with a wide range of cancer cell lines, particularly for the creation of solid tumor models. First, researchers developed 3D bioprinted cancer models by either printing immortalized cancer cells or seeding them onto a printed construct, described in this review as single cell type models or monoculture models (Table 3). As an example, Zhao et al. utilized 3D bioprinting to encapsulate HeLa cells within a gelatin-alginate-fibrinogen hydrogel to develop an in vitro model of cervical cancer. Compared to 2D cultures, the bioprinted 3D tumor cells exhibited enhanced proliferation, elevated matrix metalloproteinase expression, and increased resistance to chemotherapeutic agents [95]. Wang et al. developed a bioprinted model of glioma stem cells (GSC) to study their behavior in a 3D environment and explore their role in tumor vascularization. The study demonstrated that 3D bioprinted scaffolds offer a more physiologically relevant microenvironment that promotes GSC proliferation, stemness, and gene expression related to tumor angiogenesis [96]. To study tumor progression of hepatocellular carcinoma, Chen’s group was able to stably recapitulate the lobular architecture and the mechanical characteristics of cirrhotic liver tissue using DLP-based 3D bioprinting and a liver dECM bioink [97]. This study represents a major advancement in in vitro disease modeling, by enabling the rapid production of biomimetic architectures with tunable mechanical properties. Other examples of single cell type studies are included in Table 3.

**Table 3:** 3D bioprinted single cell type models

Cancer type	Cell lines	Matrix	Printing method	Culture time (days)	Drug testing	Comments	Ref.
Blood	MEC1	Commercialized bioink (CELLINK)	EBB	28	No	Chronic Lymphocytic Leukemia model. Transcriptome sequencing analysis showed differences in gene expression profile between 2D and 3D models.	[98]
Bone and soft tissue	MG-63	Alginate–GelMA–methylcellulose + LAP Alginate–GelMA–methylcellulose –dECM + LAP	EBB	11	Yes	Osteosarcoma model using preformed spheroids and osteosarcoma-specific bioink. Analysis of drug sensitivity compared to scaffold-free spheroids and 2D cultures.	[94]
Brain	SU3	Gelatin–alginate–fibrinogen	EBB	21	Yes	Glioma model. Analysis of gliomagenesis and sensitivity to anticancer drugs in vitro.	[11]
	Glioma stem cell GSC23	Gelatin–alginate–fibrinogen– transglutaminase	EBB	20	No	Glioma stem cells model. Biological characteristics of glioma stem cells and tumor angiogenesis.	[96]
Breast	4T1	Gelatin–alginate–dECM	EBB	7	No	Metastatic tumor model.	[73]
	MCF-7	Gelatin–alginate	EBB	9	Yes	Bioprinted model of drug-resistant breast cancer spheroid. Analysis of drug sensitivity.	[46]
	MDA-MB-231 or MCF-7	GelMA	DLP	14	Yes	Breast cancer model from xenograft tumors with blood vessel-like channels. Evaluation of the correlation between in vitro and in vivo drug screening results.	[99]
	MDA-MB-231 or MCF-7	Alginate–hydroxyapatite–periostin	EBB	21	Yes	Model of breast cancer cells seeded on 3D printed scaffolds. Comparisons of gene expression and drug sensitivity with 2D models and patient-derived scaffolds.	[100]
	ZR75.1	Gelatin–alginate	EBB	31	Yes	Breast cancer model. Comparison of drug testing in 2D model, 3D bioprinted, 3D spheroid and xenograft models.	[14]
Cervix	HeLa	Gelatin–alginate–fibrinogen	EBB	8	Yes	Cervical cancer model. Comparison of proliferation and drug sensitivity in 2D and 3D models.	[95]
	HeLa	Hydroxyethyl cellulose–alginate	EBB	7	Yes	Cervical cancer model. Analysis of cancer biology.	[101]
Colon or rectum	Caco-2	Commercialized bioink (CELLINK RGD bioink)	EBB	28	Yes	Colorectal cancer model. Histological assessment shows the formation of glandular-like structures. RNA sequencing analysis showed differences in gene expression between 2D and 3D models.	[102]
	HT-29	Gelatin–alginate (both functionalized with glycans)	EBB	14	No	Colorectal cancer model. Importance of the glycosignature in 3D bioprinted tissue models.	[62]
Head and neck	UM-SCC-12 or UMSCC-38	Gelatin–alginate–dECM from porcine tongue	EBB	19	Yes	Head and neck squamous cell carcinoma (HNSCC) model. Analysis of drug sensitivity.	[103]
Liver	HepG2	Gelatin–alginate	EBB	15	Yes	Hepatocellular carcinoma model. Comparison of gene expression and drug sensitivity in 2D and 3D models.	[104]
	HepG2	GelMA–dECM from porcine liver	DLP	7	No	Hepatocellular carcinoma model. Mimicking of clinically relevant cirrhotic liver tissue.	[97]
Lung	A549	Gelatin–alginate–fibrinogen	EBB	15	No	Lung cancer model. Comparison of gene expression and drug sensitivity in 2D and 3D models.	[105]
	A549	Alginate–HA–RGD Ca^2+^–HA	Inkjet-based	7	Yes	Lung cancer model. Cell functions in the 2D and 3D lung cancer models were evaluated by immunofluorescence staining of P-CK, ProSP-C, MUC1 and Caveolin-1.	[57]
	A549/95-D	Gelatin–alginate	EBB	28	No	Lung cancer model for studying cell invasion and migration capabilities. This model was compared to traditional 2D models.	[106]
	H358	GelMA	DLP	14	Yes	Perfusable lung cancer model. Comparison of drug sensitivity in 2D cell culture and 3D models cultured under static and dynamic conditions.	[107]

*Abbreviations*: dECM: decellularized extracellular matrix; DLP: digital light processing; EBB: extrusion-based bioprinting; GelMA: gelatin methacrylate; HA: hyaluronic acid; LAP: lithium phenyl-2,4,6-trimethylbenzoylphosphinate; RGD: arginine–glycine–aspartic acid

### Multiple cell type 3D bioprinted models

An important facet of cancer research is the structural complexity of the TME, which plays a paramount role in cancer development, progression, and therapeutic response. Tumors have been recognized as well-organized ecosystems, involving constant, dynamic, and reciprocal interactions between cancer cells and the TME [108]. The latter encompasses all the non-malignant cells in the tumor, including immune cells, fibroblasts, endothelial cells, and adipocytes, as well as non-cellular components like the ECM and soluble molecules (chemokines, cytokines and growth factors) [109, 110]. Its core is hypoxic and characterized by low extracellular pH. The ECM, consists of a large variety of macromolecules, including fibrous-forming proteins, such as collagens, laminins, proteoglycans, and glycosaminoglycans. Through direct or indirect interactions with cancer cells, stromal cells have been shown to acquire a specific biological phenotype. For instance, fibroblasts and adipocytes become respectively cancer-associated fibroblasts (CAF) [111] and cancer-associated adipocytes (CAA) [112]. To better mimic the role of neighboring cell populations and various components of the TME, additional complexity has been introduced into the bioprinted models by incorporating various cell types present in tumors (Table 4), thus allowing a better replication of the in vivo conditions present in tumors.

**Table 4:** 3D bioprinted multiple cell type models

Cancer type	Cancer cell lines	Other cell lines	Matrix	Printing method	Culture time (days)	Cell-cell interactions	Cellular migration	Vasculature	Drug testing	Ref.
Blood	K562	NK92	Gelatin–alginate	EBB	5	Yes	Yes	No	No	[59]
	HL-60	Ovarian stromal cells	Alginate–PEGylate fibrinogen	EBB	5	No	Yes	No	No	[113]
	MM1S or RPMI-8226	HS5 stromal cells	HA–alginate–PEG-DA + photoinitiator	Coaxial EBB	7	Yes	No	No	Yes	[114]
Bone and soft tissue	SW872	CAF + HUVEC	Collagen–hyaluronic acid	EBB	21	No	No	Yes	Yes	[61]
Brain	GSC23	Mesenchymal stem cells	Gelatin–alginate–fibrinogen	Coaxial EBB	5	Yes	No	No	No	[90]
	GL261	RAW 264.7 macrophages	Gelatin–GelMA	EBB	10	Yes	Yes	No	No	[115]
	Glioblastoma stem cells	Macrophages, astrocytes, hNP1 neural precursor cells	GelMA–GMHA + LAP	DLP	10	Yes	No	No	Yes	[116]
	IMR-32	HEK293 + fibroblasts	Gelatin–alginate	EBB	3	Yes	No	No	Yes	[117]
	IMR5	HUVEC	GelMA + Irgacure	EBB	14	Yes	Yes	Yes	No	[49]
	KR158B	Hematopoietic stem cells + tumor-reactive T cells (splenocytes)	Methacrylic acid comonomer– acrylamide monomer–PEG-DA –azobisisobutyronitrile–type I collagen	EBB	NA	Yes	Yes	No	No	[48]
	SK-N-AS NB	HUVEC + hMSC	GelMA–Pluronic F-127 + Irgacure	EBB	21	No	Yes	Yes	No	[118]
	SK-N-BE	CAR-T cells	GelMA + LAP	SLA	11	Yes	Yes	No	No	[119]
	SK-N-BE	L1CAM CAR-T cells	GelMA + LAP	SLA	14	Yes	Yes	No	No	[120]
	U-87	HUVEC + lung fibroblasts	Gelatin–alginate–fibrinogen	EBB	7	Yes	Yes	Yes	Yes	[58]
Breast	BT-474	HUVEC	RGD–alginate or RGD–alginate–fibrinogen	EBB	7	Yes	No	Yes	Yes	[121]
	MCF-7	ADSC	Gelatin–alginate	EBB	10	Yes	No	No	No	[122]
	MCF-7	ADSC	GelMA–alginate–dECM–collagen type I	EBB	14	Yes	No	No	Yes	[71]
	MCF-7	Dermal fibroblasts	GelMA + LAP	Inkjet-based	14	Yes	Yes	No	No	[123]
	MCF-7	HUVEC + fibroblasts	Gelatin–alginate	EBB	14	Yes	Yes	Yes	Yes	[124]
	MCF-7	CAF or breast fibroblasts + HUVEC	ColMA–HAMA–laminin-111 derived peptide IKVAV + LAP	EBB	7	Yes	No	Yes	No	[125]
	MCF-7	Fibroblasts (MRC-5)	GelMA–adipose dECM from porcine fat	EBB	7	Yes	Yes	No	Yes	[126]
	MDA-MB-231	zEGFR-CAR-NK cells	Gelatin–alginate	EBB	5	Yes	Yes	No	Yes	[59]
	MCF-7 or MDA-MB-231	Breast fibroblasts + endothelial cells (TeloHAEC)	HAMA–dECMs from porcine breast tissue + Irgacure	EBB	7	Yes	No	Yes	Yes	[68]
	MCF-7, MDA-MB-231 or CSCs	Breast fibroblasts + THP-1 monocytes	dECM from porcine breast tissue	EBB	14	Yes	Yes	No	Yes	[69]
	MDA-MB-231	ADSC or adipose spheroids	Collagen or thiolated hyaluronic acid	EBB	1	Yes	Yes	No	No	[42]
	MDA-MB-231	RAW 264.7 macrophages	Alginate	EBB	21	Yes	Yes	No	Yes	[127]
	MDA-MB-231	Engineered CD8 + T cells	Type I collagen or C2F3 matrix	EBB	NA	Yes	Yes	No	No	[45]
	MDA-MB-231, 4T1	Fibroblasts, primary limbal stem cells	dECM from caprine-origin adipose tissue	EBB	21	No	No	No	Yes	[79]
	MDA-MB-231	ADSC	HA–SH or hmHA + P(AGE-co-G) & Irgacure	EBB	21	Yes	No	No	No	[47]
	MDA-MB-231	Primary human bone marrow cells or osteoblasts	GelMA–nanocrystalline hydroxyapatite	SLA	5	Yes	Yes	No	No	[128]
	MDA-MB-231	CAF + HUVEC	Gelatin–GelMA–fibrinogen + LAP	EBB	8	Yes	No	Yes	Yes	[129]
	MF10A, MF10A-NeuN, MDA-MB-231 or MCF7	HUVEC	Gelatin–alginate or Matrigel^®^ or collagen–alginate	EBB	7	No	No	Yes	Yes	[44]
	21PT	ADSC	Gelatin–GelMA–HA–HAMA + Irgacure	EBB	24	No	No	No	Yes	[92]
Head and neck	UM-SCC-38	A8-HVFF	Gelatin–alginate–porcine dECM	EBB	22	Yes	No	No	No	[130]
Liver	SMMC-7721	Fibroblasts + HUVEC	GelMA–gelatin	EBB	14	Yes	No	Yes	Yes	[131]
	SMMC-7721	HUVEC-T1 + PBMC	Hydroxypropyl chitin–Matrigel™	EBB	7	Yes	Yes	Yes	Yes	[78]
Lung	A549	HUVEC + fibroblasts	GelMA–fibrin & PLGA shells	EBB	10	Yes	Yes	Yes	Yes	[132]
	A549	Fibroblasts	GelMA	EBB	90	Yes	No	No	Yes	[133]
	NSCLC PDX	CAF	Gelatin–alginate	EBB	25	Yes	No	No	No	[134]
Pancreas	Murine M1	CAF + splenocytes	Gelatin–alginate	EBB	NA	Yes	Yes	No	Yes	[60]
	Panc-1	CAF (MeWo) + HUVEC	Gelatin–alginate	EBB	14	Yes	No	Yes	No	[135]
Prostate	PC-3	C4-2B + fibroblasts	GelMA–CSMA–HAMA + LAP	NA	7	Yes	Yes	No	Yes	[136]
	PC-3	WPMY-1 + HUVEC	GelMA–Laponite	NA	10	Yes	Yes	NA	No	[137]
Skin	M4A4	HUVEC + fibroblasts	GelMA–fibrin & PLGA shells	EBB	10	No	Yes	Yes	Yes	[132]
	451Lu + WM164	Dermal fibroblasts	Porcine dorsal skin & human amniotic membranes	Inkjet & EBB	21	Yes	Yes	No	Yes	[138]

*Abbreviations*: ADSC: adipose-derived mesenchymal stem/stromal cells; AML: acute myeloid leukemia; A8-HVFF: immortalized human vocal fold fibroblasts; CAF: cancer-associated fibroblasts; CAR-T: chimeric antigen receptor T-cell; ColMA: methacrylated type I collagen; CSMA: chondroitin sulfate methacrylate; dECM: decellularized extracellular matrix; DLP: digital light processing; EBB: extrusion-based bioprinting; ECM: extracellular matrix; GelMA: gelatin methacrylate; GMHA: glycidyl methacrylated hyaluronic acid; HA: hyaluronic acid; HAMA: hyaluronic acid methacrylate; HA-SH: thiol-modified hyaluronic acid; hmHA: unmodified high molecular weight hyaluronic acid; hMSC: human mesenchymal stem cells; HNSCC: Head and neck squamous cell carcinoma; HUVEC: human umbilical vein endothelial cells; HUVEC-T1: simian virus 40 T antigen transfected HUVEC; LAP: lithium phenyl-2,4,6-trimethylbenzoylphosphinate; NA: not available; NSCLC: non-small cell lung cancer; P(AGE-co-G): allyl-functionalized poly(glycidol); PBMC: peripheral blood mononuclear cell; PDAC: pancreatic adenocarcinoma; PDX: patient-derived xenograft; PEG-DA: polyethylene glycol diacrylate; PLA: polylactic acid; PLGA: poly(lactic-co-glycolic) acid; RGD: arginine–glycine–aspartic acid; SLA: stereolithography

CAF promote tumor proliferation [139], drug resistance [140–142], and immune exclusion, via the excessive secretion of ECM proteins (collagens, glycoproteins, and proteoglycans) and soluble factors [143]. Secretion of Collagen I, Osteopontin, and Perlecan were observed in a bioprinted coculture model of murine pancreatic ductal adenocarcinoma cells and CAF [60]. Kort-Mascort et al. observed increased levels of collagen and matrix metalloproteinase-9 (MMP-9) over time in a coculture model of head and neck cancer cells and fibroblasts, suggesting matrix remodeling [130]. Wang’s group demonstrated that the incorporation of stromal cells in the model, such as CAF and endothelial cells, significantly promoted liposarcoma cell proliferation and invasion compared to 2D or 3D monocultures. Furthermore, the developed model successfully reproduced key in vivo features of liposarcoma, such as a soft mechanical environment, activated CAF, and an angiogenic network. Transcriptomic analysis revealed that the TME induces significant functional reprogramming [61].

Adipocyte-cancer cell crosstalk within adipocyte-rich TME, such as in breast, colorectal and ovarian cancers, drives phenotypic and functional changes that contribute to enhanced tumor progression [112] and drug resistance [144–147]. Prolonged interaction between tumor cells and adipocytes over time may also promote their differentiation into adipocyte-derived fibroblasts (ADF) which, as part of the CAF population, may be involved in tumor progression [148]. Obtaining mature adipocytes in 3D bioprinted models can however be challenging due to their fragility during the printing process, requiring maturation of immature precursor cells after printing [47], bioprinting of adipose spheroids [42]. In light of the established role of adipose tissue in breast cancer progression, Horder et al. developed a two-compartment model to evaluate the impact of adjacent adipose stroma on breast tumor cell migration. This study showed that the presence of adipose-derived stromal cells increased several key migration parameters of breast cancer cells, including the motile fraction, persistence, invasion distance, and speed [137].

Some authors have also used these models to characterize immune-cancer cell interaction, as well as the mobility and antitumor activity of immune cells [59, 60, 115–127]. A 3D bioprinted breast cancer model cocultured with T cells suggests a paracrine mode of immune–cancer interaction when the T cells are bioprinted proximal to the tumor [45]. Another study showed that the addition of hematopoietic stem cells (HSC) in murine glioma model increased tumor-reactive T cell migration in concordance with previous in vivo studies [48].

Cancer metastasis accounts for a large number of cancer-related deaths and presents a significant challenge to effective treatment. Research groups have developed coculture models in an attempt to reconstitute an in vitro metastatic setting [138–149]. Using inkjet-based and pneumatic extrusion-based bioprinting technique, Vásquez-Aristizabal et al. developed a tri-layered melanoma model. To replicate the dermal, epidermal, and basement membranes of the skin, three different bioinks were engineered using dermis and epidermis-derived dECM, as well as human-derived amniotic membrane. These bioinks were used to construct a layered model organized from top to bottom into a tumor compartment containing two melanoma cell lines, a cell-free layer mimicking the basement membrane, and a healthy dermal compartment populated with human dermal fibroblasts. This setup enables the reconstitution of metastatic processes toward the healthy dermis [138]. Another study used SLA to fabricate a bioprinted breast cancer bone metastasis model [128]. Different bone matrices consisting of osteoblasts or bone marrow mesenchymal stem cells (MSC) embedded in GelMA hydrogel containing nanocrystalline hydroxyapatite (nHA) have been developed. A limitation of tumor migration studies performed under static conditions is that they fail to recapitulate the complex migratory environment, which includes vascularized and lymphatic tissue. The implementation of microfluidic systems enables the controlled study of migration under flow, and holds therefore great promise for in vitro metastasis models.

Abnormal vasculature is a key feature of solid tumor. Tumor blood vessels are disorganized, inconsistent, and twisted, frequently lacking smooth muscle, innervation, and intact endothelial or basement membrane layers [150]. To mimic tumor vasculature, researchers first integrated endothelial cells into bioinks [44, 68]. For instance, Han et al. were able to form microvessels by bioprinting endothelial cells and fibroblasts in a gelatin–alginate–fibrinogen bioink [58]. However, most current 3D bioprinted cancer models fail to integrate functional blood and lymphatic vessels, which are essential for delivering oxygen and nutrients to tumors and for disseminating cancer cells, respectively. Therefore, 3D models with perfusable vascular channels have been developed [151]. As an example, Ning et al. successfully developed a neuroblastoma model with endothelialized bioprinted microchannels that enable in vitro dynamic culture [49]. Perfusion allows the culture medium to enter through the channels and penetrate the GelMA chamber housing the neuroblastoma spheroids, reproducing a vessel perfusing a solid tumor. At the interface regions, endothelial cells showed an expansive penetration deep within the cancer spheroids, accompanied by evidence of new capillary formation, indicating crosstalk between neuroblastoma cells and endothelial cells.

Integrating 3D bioprinting with microfluidic technology [152], 3D bioprinted cancer-on-chip platforms [78, 121, 153] are emerging to create anatomically scaled 3D models with spatiotemporally controlled cell distribution and physiologically relevant fluid flow [154]. Das et al. developed a 3D bioprinted lung cancer-on-a-chip model that replicates the human lung’s physical, mechanical, and biological properties [133]. A549 cells and fibroblasts were bioprinted onto polydimethylsiloxane (PDMS) fluidic channels using GelMA hydrogel. These fluidic channels allow the circulation of cell culture media or air flow, creating a pseudo-respiration including exhalation/inhalation to mimic the in vivo microenvironment.

These multicellular models can recapitulate key elements of the TME, including complex cellular architectures, tunable matrix compositions, as well as specific mechanical and biochemical characteristics, such as stiffness and cytokine gradients [22, 44, 155]. Furthermore, they are able to mimic oxygen and pH gradients, which are notably present in the majority of solid tumors [156].

### Bioprinted models for drug screening

Current in vitro anticancer drug screening models have significant limitations and consequently tend to overestimate drug sensitivity, providing limited predictive value for clinical outcomes. Therefore, a common use of 3D bioprinted models applies to drug screening (Table 5), either in the single cell type or multiple cell type models. Different anticancer therapeutics have been tested, including small molecules such as oxaliplatin, doxorubicin, and 5-fluorouracil, as well as antibodies (e.g., anti-CTLA4 antibody and metuzumab). Some researchers have shown that cellular-based therapies, such as chimeric antigen receptor T (CAR-T) cells and CAR-NK cells, can also be screened using 3D bioprinted models.

**Table 5:** Evaluation of drug sensitivity in 3D bioprinted cancer models

Cancer model	Tumor cell line	Other cell types	Drug tested	Drug application timepoint after printing (days)	Drug exposure time	Read-out	Conclusions	Ref.
Blood	MM1S or RPMI-8226	HS5 stromal cells	Bortezomib, tocilizumab	NA	NA	Fluorescence microscopy	3D model was less sensitive. HS5 stromal cells conferred resistance.	[114]
Bone and soft tissue	MG-63	-	Doxorubicin	1	72 h	- Fluorescence microscopy after live/dead cell staining - Flow cytometry analysis after Annexin/PI staining - AlamarBlue™ assay	3D bioprinted model was less sensitive compared to scaffold-free spheroids and 2D models.	[94]
	SW872	CAF + HUVEC	Doxorubicin	NA	48–72 h	Luciferase assay (Bright-Glo™)	3D model was less sensitive. 3D co-culture model with CAF only or CAF + HUVEC further increased the survival rate of SW872 cells.	[61]
Brain	Glioblastoma stem cells	Macrophages, astrocytes, hNP1 neural precursor cells	Erlotinib, gefitinib, temozolomide, abiraterone, vemurafenib, ifosfamide	5	72 h	CellTiter Glo^®^	3D model was less sensitive than spheres. M2 macrophages reduced sensitivity.	[116]
	SU3	-	Temozolomide	NA	48 h	AlamarBlue™ assay	3D model was less sensitive (~ 2-fold).	[11]
	U-87	HUVEC + lung fibroblasts	Temozolomide, sunitinib	NA	72 h	Actin/DAPI staining	Endothelial cells enhanced the potency of the temozolomide + sunitinib combination.	[58]
Breast	MCF-7	-	Camptothecin, paclitaxel	5	48 h	Fluorescence microscopy	3D model was less sensitive.	[46]
	MCF-7	-	Drug-loaded nanoparticles (coumarin 6 or docetaxel)	7	6 h	- Confocal microscopy - Flow cytometry	3D model was less sensitive.	[70]
	MCF-7	-	Doxorubicin	7	48 h	AlamarBlue ^TM^ assay	3D model was less sensitive.	[71]
	MDA-MB-231 or MCF-7	-	Doxorubicin, capecitabine	NA	NA	MTS assay	For capecitabine: 3D models containing channels showed lower IC50 values than those without.	[99]
	MDA-MB-231 or MCF-7	-	Doxorubicin, 5-fluorouracil	21	48 h	- qPCR analysis - lactose dehydrogenase release	Cells cultured on 3D printed and patient-derived scaffolds were shown to respond similarly to cytotoxic drug treatments.	[100]
	MDA-MB-231	-	CAR-NK cells	NA	24 h	Flow cytometry	Cytotoxic effect of the zEGFR-CAR-NK on cancer cells.	[59]
	ZR75.1	-	Rapamycin, doxycycline, doxorubicin	3, 7 or 10	72 h	AlamarBlue ^TM^ assay	3D bioprinted model share highest similarity with xenograft model.	[14]
	BT-474	HUVEC	Doxorubicin	7	24 h	Fluorescence microscopy	3D bicompartimental coculture model was less sensitive than 3D monoculture and 3D mixed coculture models.	[121]
	MCF-7	HUVEC + fibroblasts	Doxorubicin, paclitaxel, BEZ235, gemcitabine, sunitinib	4	72 h	CellTiter Glo^®^	3D model was less sensitive: 20-fold and ~ 5000-fold more resistance to doxorubicin and paclitaxel, respectively.	[124]
	MCF-7	CAF + HUVEC	Paclitaxel	4	72 h	Ki67 immunostaining	CAF protected against radiotherapy.	[125]
	MCF-7	Fibroblasts (MRC-5)	Oxaliplatin, doxorubicin, 5-fluorouracil, gemcitabine	NA	48 h	CCK-8 assay	CAF reduced drug sensitivity.	[126]
	MDA-MB-231 or MCF-7	Fibroblasts + endothelial cells (TeloHAEC)	Tamoxifen, doxorubicin, paclitaxel	1	72 h	- Fluorescence microscopy after live/dead cell staining - LDH assay	Use of model as drug screening platform.	[68]
	MDA-MB-231	RAW 264.7 macrophages	Gefitinib, zoledronic acid, Rac 1 inhibitor II	0	7 days	Fluorescence microscopy	Multi-compartment breast cancer model showing a dose-dependent modulation of cell migration when treated with clinical drugs.	[127]
	MDA-MB-231	HUVEC	Paclitaxel	NA	NA	- Resazurin assay - Fluorescence microscopy	Endothelial cells protected individually printed tumor cells.	[44]
	MDA-MB-231	CAF + HUVEC	Doxorubicin	NA	72 h	CellTiter Glo^®^	3D models showed a significant higher metabolic activity.	[129]
	MDA-MB-231	Dermal fibroblasts + HUVEC	Doxorubicin, CAR-T cells	3	24–72 h	- Fluorescence microscopy - AlamarBlue™ assay	Dose-dependent cytotoxic effect of doxorubicin.	[43]
	MDA-MB-231, 4T1	Fibroblasts, primary limbal stem cells	5-fluorouracil	3	72 h	Fluorescence microscopy	Dose-dependent cytotoxic effect of 5-fluorouracil.	[79]
	MF10A, MF10A-NeuN, MDA-MB-231 or MCF7	HUVEC	Paclitaxel	NA	NA	- Resazurin - Live/dead assay	Cells printed as spheroids were less sensitive than individual cells, but this effect was abrogated in coculture with endothelial cells.	[44]
	MCF-7, MDA-MB-231 or MDA-MB-231 isolated CSCs	Breast fibroblasts, THP-1 monocytes	Doxorubicin, paclitaxel, docetaxel, reparixin	3	72 h	LDH assay	Antimitotics exhibited low cytotoxicity in the bioprinted model, unlike reparixin.	[69]
	21PT	ADSC	Doxorubicin	21	72 h	Staining with cleaved Caspase-3 HPLC to evaluate drug concentration within constructs	3D model was less sensitive at low doses.	[92]
Cervix	HeLa	-	Paclitaxel	5	72 h	CCK-8 assay	3D model was less sensitive (~ 8-fold).	[95]
	HeLa	-	Paclitaxel	3	48–96 h	NA	3D model tends to be more sensitive (several-fold).	[101]
Colon or rectum	Caco-2	-	5-fluorouracil, oxaliplatin, irinotecan	12	72 h	MTT assay	3D model was less sensitive: ~2.4-fold and ~ 6-fold more resistance to irinotecan and 5-fluorouracil, respectively.	[102]
Head and neck	UM-SCC-12 or 38	-	Cisplatin, 5-fluorouracil	7	7 days	WST-1 proliferation assay	3D model was less sensitive.	[103]
	UM-SCC-14 C or 11B or 22B	-	Cisplatin	6	-	Cell Titer Glo^®^	3D model enabled drug response.	[157]
Liver	HepG2	-	Cisplatin, sorafenib, regorafenib	7	72 h	CCK-8 assay	3D model was less sensitive.	[104]
	SMMC-7721	Fibroblasts + HUVEC	Cisplatin, doxorubicin, luteolin	14	24–48 h	- Fluorescence microscopy - CCK-8 assay	3D model enabled drug response.	[131]
	SMMC-7721	HUVEC-T1 + PBMC	Metuzumab	5	48 h	CCK-8 assay	Antibody-dependent cell-mediated cytotoxicity (ADCC) exihibted higher effectiveness in the microfluidic model than in 3D printing models.	[78]
Lung	A549	-	Doxorubicin, 5-fluorouracil, paclitaxel, magnesium methyl carbonate, cisplatin, osimertinib, afatinib, gefitinib, erlotinib	1	48 h	CCK-8 assay	3D model was less sensitive, particularly to EGFR inhibitors.	[57]
	H358	-	Gemcitabine	14	4 days	- XTT assay - EdU assay - Live/dead assay	3D model was less sensitive: ~3-fold. Under dynamic cultivation, the drug-mediated apoptosis was increased.	[107]
	A549	Fibroblasts	Paclitaxel	5	48 h	Fluorescence microscopy after live/dead cell staining	Dose-dependent response.	[133]
Nerve	IMR-32	HEK293 + fibroblasts	Panobinostat, blasticidin	1	24, 48–72 h	- Fluorescence microscopy - XTT assay for monocultures	Differences in sensitivity between 2D and 3D cultures were cell-type specific.	[117]
Pancreas	Murine M1	CAF + splenocytes	Anti-CTLA4 antibody	0.5	48 h	Live imaging	Rapid homing of immune cells toward the cancer cells.	[60]
Prostate	PC-3	C4-2B + fibroblasts	Docetaxel	7	48 h	CCK-8 assay	3D model was less sensitive.	[136]
Skin	451Lu + WM164	Dermal fibroblasts	PLX4720, Vemurafenib (PLX4032)	1	1, 2, 3 or 7 days	- CellTox™ green cytotoxicity assay - Confocal laser scanning microscopy	PLX4720 and vemurafenib were cytotoxic in a complex skin mimicking-environment.	[138]

*Abbreviations*: CAF: cancer-associated fibroblasts; CCK-8: Cell Counting Kit-8; EdU: 5-ethynyl-2′-deoxyuridine; EGFR: epidermal growth factor receptor; HUVEC: human umbilical vein endothelial cells; HUVEC-T1: simian virus 40 T antigen transfected HUVEC; NA: not available; PBMC: peripheral blood mononuclear cell; qPCR: real-time polymerase chain reaction; TeloHAEC: human aortic endothelial cells

To observe cytotoxicity, different approaches can be used, ranging from microscopy [68, 94, 114] to metabolic activity assays, such as AlamarBlue™ [11, 14, 94] and Cell Counting Kit-8 (CCK-8) assays [57, 104, 126]. However, there are several limitations. Because bioprinted models consist of multiple layers, certain microscopy techniques, such as confocal microscopy, can only image cells up to 100 μm deep. Additionally, metabolic activity assays do not allow for differentiation of cancer cells from stromal cells in a coculture setting. The results should also be interpreted with caution, as they rely heavily on homogeneous distribution of the reagent throughout the cells, which may not occur in large spheroids within the 3D bioprinted models.

The effectiveness of anticancer drugs in treating solid tumors depends on the drug’s ability to reach all cancer cells in the tumor. Overall, 3D monocultured cells are less sensitive to certain antineoplastic drugs than cells grown in 2D monolayer systems [11, 46, 57, 94, 95, 103, 104]. For example, 3D bioprinted models of MCF-7 cells exhibited higher IC50 values following doxorubicin treatment than 2D cultures, due to cell-cell and cell-ECM interactions present in the 3D model [71].

Stromal cells, vascular endothelial barrier, and ECM are essential components that can affect drug transport to cancer cells. For instance, changes in the ECM composition may influence drug responses by altering local drug availability, affecting the expression of drug targets, or changing intrinsic cellular defense mechanisms, such as increased repair of DNA damage or evasion of apoptosis [158]. A liposarcoma model integrating CAF and endothelial cells exhibited greater resistance to the standard chemotherapeutic agent doxorubicin than 2D or 3D monocultures. These results suggest that chemoresistance is caused by physical barriers resulting from cell-matrix remodeling and protective paracrine signaling from stromal cells [61].

A study showed that 3D bioprinted constructs with breast cancer cells surrounded by adipose-derived mesenchymal stem cells (ADSC) exhibited reduced cleaved caspase-3-positive cells when exposed to a low dose of doxorubicin. After increasing the thickness of ADSC layers to mimic the state of obesity, they demonstrated lower apoptosis after doxorubicin treatment. Furthermore, the addition of a lysyl oxidase (LOX) inhibitor decreased the stiffness in the ADSC region but not the breast cancer region, and it enhanced doxorubicin sensitivity of the breast cancer cells [92]. This work presents evidence that 3D bioprinted models can represent in vivo situations that can be used for drug testing [159].

To replicate glioblastoma tumor mass infiltration by macrophages and microglia, Tang et al. used DLP to fabricate 3D tetra-culture glioblastoma tissue models. Adding macrophages to the bioprinted model increases the resistance of glioblastoma stem cells to epidermal growth factor receptor (EGFR) inhibitors [116]. A study by Mazzaglia et al. found that splenocytes could infiltrate 3D bioprinted cancer models, and that their motility increased following the addition of an anti-cytotoxic T lymphocyte-associated protein-4 (CTLA-4) antibody [60]. In 2021, Künkele’s group demonstrated that 3D bioprinted models enable the detection and quantification of CAR-T cell tumor infiltration [119]. Using the same 3D bioprinted model, they recently elaborated a strategy to identify genetic factors that drive CAR-T cell migration into and through a tumor mass [120], highlighting the relevance of 3D bioprinted models for initial screening. In 2023, Kim et al. developed a 3D bioprinted model for CAR-NK cell therapy. 3D bioprinted CAR-NK cells cultured with 2D MDA-MB-231 cells were able to leave the 3D model and lyse MDA-MB-231 cells [59].

To better mimic drug exposure, Dey et al. developed a dynamic-flow based 3D bioprinted vascularized breast tumor model responding to chemotherapy and CAR-T cell-based immunotherapeutics [43]. Therefore, accurate 3D cell systems could improve the predictability of sensitivity of cancer treatments. However, few comparisons have been performed between 3D models and in vivo sensitivity [14].

When evaluating drug efficacy, it is important to consider the delivery of substances across the vascular endothelial barrier to tumor masses. Therefore, combining 3D bioprinting and microfluidics, Lee et al. propose a bioprinted, multi-composition array-on-a-chip, enabling high throughput drug screening. A multivariable analysis was conducted in 36 models bioprinted on a single microfluidic device, including triplicates of three model compositions (BT-474 monoculture, single-compartment coculture of BT-474 and HUVEC, and two-compartmental coculture of BT-474 and HUVEC), treated at four doxorubicin drug concentrations. The two-compartmental coculture model showed BT-474 spheroids in the center surrounding by a vascular endothelial barrier formed by HUVEC cells. After doxorubicin treatment, the BT-474 monoculture model exhibited smaller BT-474 spheroids compared to the other two models, while the separated BT-474–HUVEC model displayed the slowest drug penetration, the highest viability, and the highest IC50 values [121]. This highlights the role of the TME, in particular the HUVEC barrier, in hindering molecular diffusion.

Multicellular heterogeneity, matrix composition and stiffness, oxygen/nutrient gradients, and vasculature are key features of the TME that directly affect treatment response. Therefore, drug testing results are highly influenced by the choice of cells (primary or commercialized), cell density, bioink composition, culture duration, and drugs tested, resulting in different IC50 values depending on the model used. Further development is necessary for the systems to achieve high model complexity that is representative of human physiology, and high experimental throughput.

## Bioprinted patient-derived model for drug screening

To more closely mimic clinically relevant tumors and recapitulate the heterogeneity of patients, models directly derived from primary patient samples are increasingly being used. As indicated in the introduction, PDX have shown their ability to partially replicate the patient’s tumors, yet may be lengthy to establish and, in some cancer cell types, difficult to obtain with a long time to read-out and a high associated cost. Alternatives include patient-derived organoids (PDO) [160–162] and patient-derived explants (PDE) [163], which have a low success rates and limited viability timeframes, respectively. In several situations, there are no specific recommendations regarding the most effective treatment sequence to follow in the presence of metastatic disease or relapse, nor biomarkers allowing to choose among several therapeutic alternatives. Therefore, new preclinical models are needed to guide individualized medicine.

Bioprinting allows the analysis of patient-derived samples over a short period of time, ranging from a few days to several weeks, making them compatible with personalized medicine approaches. For instance, Du et al. propose a bioprinted model of patient-derived gastrointestinal stromal tumors that is ready within 10 days post-surgery, to provide individualized treatment recommendations [164].

Several efforts are ongoing to characterize which cells survive or thrive in a bioprinted environment. Currently available studies (Table 6) suggest that 3D bioprinted fresh tumor samples yield interesting results in terms of interactions with cells from the TME, as well as for drug sensitivity screening. Recently, Du et al. demonstrated that their patient-derived bioprinted gastric cancer models retained the original tumor characteristics and showed a significant correlation in drug sensitivity between the 3D models and the clinical efficacy observed in patients [165]. Using DLP technology and a GelMA–HAMA bioink, Liu et al. established patient-derived 3D thymic epithelial tumor (TET) models reproducing histological architecture, genomic profile, and mechanical stiffness of primary TET tumors. Using these models, they were able to identify lurbinectedin as a potent therapeutic candidate that exerts significant regulatory effects on tumor stress responses and microenvironmental remodeling. Furthermore, additional analysis revealed several genes that are associated with efficacy and have strong prognostic potential. This illustrates how 3D bioprinted cancer models can facilitate the evaluation of drug responses and the identification of translational biomarkers [166].

**Table 6:** 3D patient-derived bioprinted cancer models

Cancer type	Matrix	Printing method	Culture time (days)	Proliferative capacity	Drug testing	Comments	Ref.
Blood	Commercialized bioink (CELLINK)	EBB	28	Around 40% viable cells at day 28	No	Chronic Lymphocytic Leukemia model. Transcriptome sequencing analysis showed difference in gene expression profile between 2D and 3D models.	[98]
Brain	Gelatin–alginate or Pluronic F-127	EBB	35	Yes	Yes	Microengineered perfusable 3D bioprinted glioblastoma model. The 3D model showed growth curves, drug response, and genetic signatures similar to those of glioblastoma cells grown in orthotopic mouse cancer models, as opposed to 2D culture.	[167]
	dECM or collagen	EBB	14	Yes (CCK-8 assay)	Yes	Glioblastoma-on-chips. The model showed differential responses that aligned with clinical outcomes and patient-specific sensitivity to potential drug combinations.	[168]
Colon or rectum	Gelatin–alginate	EBB	14	Yes (Ki67 staining)	Yes	Colorectal cancer and liver metastases. Drug response to neoadjuvant chemotherapy in bioprinted models correlates with clinical outcomes.	[169]
	GelMA	Inkjet-based	NA	NA	Yes	Patient-derived bioprinted models of colorectal cancer and healthy organoids. Correlation between organoid invasion speed and normalized spreading speed of the paired patients.	[170]
	Gelatin–alginate (functionalized with glycans)	EBB	21	NA	No	Colorectal cancer model. Analysis of glycan signaling.	[62]
	Commercialized bioink (CELLINK RGD bioink)	EBB	14	NA	No	Colorectal cancer model. Histological analysis showed clusters of cells with malignant histopathological appearance and expression of epithelial cell surface markers.	[102]
Head and neck	TEMPO-NC or carboxy-NC or GelMA–alginate	EBB	10	NA	Yes	Compared to the spheroid models, the cytotoxic effects were more reflecting the response in patients.	[157]
Liver	Gelatin–alginate–Matrigel™	EBB	7	Yes	Yes	Intrahepatic cholangiocarcinoma model. The bioprinted cells were observed to have a high survival rate, active proliferation, and colony-forming ability.	[75]
	Gelatin–alginate	EBB	28 (80%)	Proliferation rate: between 10 and 90%	Yes	Hepatocellular carcinoma model for drug screening. This model retained the features of parental hepatocellular carcinoma model, including stable expression of biomarkers, sgenetic alterations and expression profiles.	[171]
Lung	GelMA–PEG-DA	SLA	6	Yes	No	Model of non-pretreated early-stage NSCLC. The bioprinted model was better to mimic tumor complexity and regulate cancer cell behavior than 2D culture models.	[172]
	GelMA–HAMA + LAP	DLP	5	Yes	Yes	This model effectively replicates key features of the original tumor.	[173]
Pancreas	Gelatin–alginate	EBB	18	Prolonged viability	Yes	This model maintains high-fidelity histological and genomic characteristics. Chemotherapeutic drug sensitivity in bioprinted models correlates with clinical outcomes.	[174]
	Gelatin–alginate	EBB	10	Yes (Ki67 staining)	Yes	Two-compartment model. The primary cells retained E-cadherin+ cell-cell junctions and proliferative capacity, as determined by Ki67 + staining.	[124]
Stomach	Gelatin–alginate–Matrigel™	EBB	63	Yes (Ki67 staining)	Yes	This study showed that patient-derived gastric adenocarcinoma cells can be reprinted for at least three 21 days culture cycles following bioprinting.	[76]
	GelMA–HAMA + LAP	Lithography	15	Yes (Ki67 staining)	Yes	The models preserved the histological architecture, biomarker expression abundance and genetic mutation profiles of the parental tumors. A significant correlation was observed in drug sensitivity between the 3D models and the clinical efficacy observed in patients.	[165]
	Stomach-dECM	EBB	10	Yes	Yes	Bioprinted vascularized organoid model show a functional endothelium and PDO-dependent sprouting behavior.	[50]
	GelMA–HAMA + LAP	EBB	14	Yes (CellTiter-Glo^®^)	Yes	This model recapitulates the histological architecture, biomarker expression, and molecular features of their corresponding parental tumors.	[164]
Thymus	GelMA–HAMA + LAP	DLP	15	Yes	Yes	Compared to organoids, 3D thymic epithelial tumor models, provide higher morphological fidelity and cellular consistency.	[166]

*Abbreviation*: carboxy-NC: carboxymethylated nanocellulose; CCK-8: Cell Counting Kit-8; dECM: decellularized extracellular matrix; DLP: digital light processing; EBB: extrusion-based bioprinting; GelMA: gelatin methacrylate; HAMA: hyaluronic acid methacrylate; LAP: lithium phenyl-2,4,6-trimethylbenzoylphosphinate; NA: not available; NSCLC: non-small cell lung cancer; PDO: patient-derived organoids; PEG-DA: polyethylene glycol diacrylate; RGD: arginine–glycine–aspartic acid; SLA: stereolithography; TEMPO-NC: TEMPO-mediated oxidized nanocellulose

Combining PDO and 3D bioprinting, Kim et al. replicate clinical outcomes that are specific to gastric cancer subtypes. After printing a frame with two compartmentalized chambers using polyethylene-co-vinyl acetate (PEVA), they printed a Pluronic F-127 hydrogel in one of the chambers as a tubular shape, and a PDO-laden stomach-dECM in the opposite chamber. After polymerization of the printed construct, the Pluronic F-127 hydrogel was removed, and human umbilical vein endothelial cells (HUVEC) were seeded in the hollow channel. Using this setting, the authors emphasize the importance of proximity between tumor cells and blood vessels to promote angiogenic signals. Furthermore, they assessed the sprouting behavior of three different cancer subtypes and observed distinct angiogenic responses, making this model a valuable tool for evaluating the angiogenic potential of individual patient tumors and for generating vascularized cancer models. Finally, they were able to demonstrate that their model elicits patient-specific transcriptomic features and can predict responses to VEGFR2-targeted therapy in diffuse-type gastric cancers. This model represents a significant advancement in creating preclinical models that more closely represent tumor heterogeneity, facilitating precision cancer medicine and improving patient outcomes [50]. Yi et al.‘s work demonstrated the applicability of cancer-on-a-chip model to patient-derived cells, reflecting biochemical and biophysical cues observed in a native glioblastoma. This model produced differential responses that aligned with clinical outcomes following concurrent chemoradiation with temozolomide. Additionally, it demonstrated patient-specific sensitivity to potential drug combinations [168]. These results suggest that an ex vivo system could be used to model personalized cancer treatments and guide clinical decisions to overcome refractory cancers.

Before implementing patient-derived bioprinted models in the clinic, their predictive value for anticancer drugs must be further evaluated. For this purpose, different observational studies have been registered on ClinicalTrials.gov (NCT04755907, NCT06792149, NCT06121622). The results of the NCT04755907 clinical trial (registered on February 16, 2021) have been published [169]. This study enrolled 40 patients undergoing surgery for colorectal cancer and 31 patients who underwent colorectal liver metastasis surgery, from which 37 and 29 bioprinted models were established, respectively. This study showed that the bioprinted models maintained the specific biomarkers and characteristic mutation profiles of the corresponding fresh tumors. Furthermore, drug response data from the bioprinted models showed a significant correlation with the clinical outcomes of patients after receiving neoadjuvant chemotherapy. Thus, available data suggest that 3D bioprinting of fresh samples is a useful tool to evaluate correlations between in vitro sensitivity and patient outcome in series of homogeneously treated patients. This is promising for the screening of novel agents which have not yet reached the clinic.

There is great hope that, in the future, patient-derived bioprinted cancer models could serve as a reliable and highly efficient platform for personalized cancer treatment. However, many challenges remain to be addressed to use this approach as a routine method for treatment selection at the individual level. First and foremost, robust methods for model establishment will need to be standardized to obtain both high yields and models which are representative of the primary tumor. In some instances, there might indeed be a significant limitation in the total number of cells required for bioprinted models, once routine diagnostic procedures have been performed. In our experience, a close collaboration between the pathology laboratory and the bioprinting facility constitutes an essential logistical aspect [175]. Furthermore, a single tumor specimen cannot suffice to analyze tumor heterogeneity, with the risk that other areas of the tumor or other localizations may present a different drug sensitivity profile. Moreover, the fraction of proliferative tumor cells in the bioprinted models may be relatively low as compared to what is classically observed with cell lines. While this may not be an issue with immunotherapeutic approaches which destroy tumor cells irrespective of their proliferative status, it may prove to be problematic to select for agents which target dividing cells. Importantly, there will be a need to standardize sensitivity criteria in bioprinted models. In vitro models will provide data akin to inhibitory concentration 50% or 90% values, and these will need to be correlated with the quality of response data in patients receiving the same compounds. Additionally, there will also be a need to evaluate combination regimens in bioprinted models, which will be technically challenging given the limited number of experimental replicates which can be produced from a given clinical sample. Altogether there remains a significant number of issues to address to demonstrate feasibility and translational relevance of personalized drug screening.

## Conclusions

Preclinical cancer modeling applied to drug screening remains a difficult endeavor. While the selection of potent candidates from a library of molecules seems increasingly feasible, the therapeutic adaptation in an individual patient will require to overcome several technical hurdles. Key issues relate to relevance of the model to the clinic, tumor diversity, intrapatient tumor heterogeneity and relevance of the read-out. As opposed to 2D models, 3D models offer the possibility to have a more clinically relevant proliferative fraction, thus avoiding false positive during drug screenings. Patient-derived bioprinted models offer an interesting alternative but their proliferation fraction may not always be compatible with the time line required for drug adaptation. Tumor heterogeneity during drug screening can be at least partly taken into account by diversifying the number of models tested, a strategy that is increasingly being applied to PDX models. Interestingly, 3D bioprinting can be applied to dissociated PDX tumors (personal data) or PDO, offering the possibility to screen a large number of models at a reduced cost.

Among available 3D models, 3D bioprinting has clearly evolved as an interesting alternative model to study cancer cell biology and sensitivity to therapy. Among the main advantages are the technical simplicity of multiple replicates, the high reproducibility, the possibility to print the majority of cell lines currently used in 2D, and the ability to mimic primary tumors by printing material provided by fresh tumor samples. Additionally, various analytical techniques may be applied, such as continuous methods (e.g., live imaging and metabolic activity assays) or terminal read-outs (flow cytometry, light sheet microscopy, immunochemistry, and spatial transcriptomics).

In spite of the development of microperfused systems, aiming to mimic the presence of a tumor microvasculature and better reproduce dynamic drug exposure, these models remain complex and have not yet reached a high-throughput stage [78]. A physiologically complete model should include not only input of therapeutic molecules or cells but also their elimination through the equivalent of vascular or lymphatic channels.

Complexification of 3D models using cellular components of the TME has shown its potential to better apprehend its role on tumor cell biology and drug sensitivity. These models could help overcome current challenges in cancer treatment, such as the development of personalized tests to predict therapy response, and the identification of new drugs for cancers without standardized treatment regimens. However, there is presently a trade-off between model complexification, resulting in improved relevance, and its use as a drug screening tool. An additional hurdle of multicellular models is related to the fact that they are currently composed of cell types originating from several donors, which raises the issue of allogeneic interactions. Obtaining autologous samples such as blood, fibroblasts or adipocytes from patients to match their tumors has proven to be logistically complicated. Alternatives may be to derive mature cells from autologous stem cells [176].

An intriguing issue is whether resistant models developed in the 3D context will better mimic resistance observed in vivo [177], as opposed to the phenotypes of resistance models developed in 2D systems [178, 179]. Given the importance of microenvironmental cues in the development of resistance phenotypes, it will be interesting to compare resistant models developed in 2D, 3D and in vivo systems.

A promising advancement is the development of stimulus-responsive materials, which enable spatiotemporal transformations of 3D constructs in response to external cues. Extracellular chemical gradients can be dynamically manipulated using 3D-printed capsules, functionalized with nanomaterials and sensitive to stimuli, containing growth factors, enabling post-printing cellular modulation. Tumor cell migration can be effectively directed using 3D-printed programmable release capsules that serve as dynamic sources of chemo-attractants (e.g., epidermal growth factor (EGF)) [132]. Additionally, a programmable pulsatile drug release system using laser-triggered release capsules has been reported [180].

The current state of the art provides a diversity of 3D models, each with its advantages and limitations. In the field of cancer drug screening, a possible pipeline could consist in the sequential filtering of candidate compounds in conventional 2D systems using tumor cell lines, followed by relatively cheap and rapid analysis of spheroids, then completed by more complex organoid or 3D bioprinted models representative of tumor diversity in patients. Animal models would then be reserved to strictly selected agents with a higher probability of success in vivo. Preclinical drug screening tools have considerably evolved over the past decades and the role of 3D bioprinted models will require both technological developments and comparative studies in the years to come.

## Supplementary Information

Below is the link to the electronic supplementary material.


Supplementary Material 1


## Data Availability

No datasets were generated or analysed during the current study.

## Abbreviations

2D: Two-dimensional
2PP: Two-photon polymerization
3D: Three-dimensional
A8-HVFF: Immortalized human vocal fold fibroblasts
ADF: Adipocyte-derived fibroblasts
ADSC: Adipose-derived mesenchymal stem/stromal cells
AML: Acute myeloid leukemia
CAA: Cancer-associated adipocytes
CAD: Computer-aided design
CAF: Cancer-associated fibroblasts
Carboxy-NC: Carboxymethylated nanocellulose
CAR-T: Chimeric antigen receptor T-cell
CDX: Cell line-derived xenograft
ColMA: Methacrylated collagen
CSMA: Chondroitin sulfate methacrylate
CCK-8: Cell Counting Kit-8
DEAE: Diethylaminoethyl
DLP: Digital light processing
dECM: Decellularized extracellular matrix
EBB: Extrusion-based bioprinting
ECM: Extracellular matrix
EdU: 5-ethynyl-2′-deoxyuridine
EGF: Epidermal growth factor
EGFR: Epidermal growth factor receptor
FDM: Fused deposition modeling
GelMA: Gelatin methacrylate
GSC: Glioma stem cells
HSC: Hematopoietic stem cells
HA: Hyaluronic acid
HAMA: Hyaluronic acid methacrylate
HA-SH: Thiol-modified hyaluronic acid
hmHA: Unmodified high molecular weight hyaluronic acid
hMSC: Human mesenchymal stem cells
HNSCC: Head and neck squamous cell carcinoma
HTS: High throughput screening
LAB: Laser-assisted bioprinting
LAP: Lithium phenyl-2,4,6-trimethylbenzoylphosphinate
LIFT: Laser-induced forward transfer
LOA: Likelihood of approval
LOX: Lysyl oxidase
MMP-9: Matrix metalloproteinase-9
NA: Not available
NSCLC: Non-small cell lung cancer
P(AGE-co-G): Allyl-functionalized poly(glycidol)
PBMC: Peripheral blood mononuclear cell
PCL: Polycaprolactone
PDAC: Pancreatic adenocarcinoma
PDE: Patient-derived explants
PDO: Patient-derived organoids
PEG: Polyethylene glycol
PEG-DA: Polyethylene glycol diacrylate
PDX: Patient-derived xenografts
PLA: Polylactic acid
RGD: Arginine–glycine–aspartic acid
SLA: Stereolithography
TeloHAEC: Human aortic endothelial cells
TEMPO-NC: TEMPO-mediated oxidized nanocellulose
TME: Tumor microenvironment
qPCR: Real-time polymerase chain reaction
