# Supplementary material for: 3D bioprinting for cancer modeling and drug screening
Source: Biomark Res. 2026 Jun 20;14:105. doi: 10.1186/s40364-026-00951-x (PMC13536750; doi:10.1186/s40364-026-00951-x)
Supplement: Supplementary file 1 [file 40364_2026_951_MOESM1_ESM.docx]

**Supplementary figure 1. The three steps of the 3D bioprinting process: preprocessing, processing, and postprocessing.**

The preprocessing consists of the computer-aided design (CAD) modeling of the 3D model. During the bioprinting process, the bioink is prepared and then ejected/extruded using a bioprinter to form the desired 3D models. Finally, in order to maintain the structural integrity and stability of the bioink after printing, the 3D structures must be crosslinked. After X days in culture, the 3D bioprinted models can be characterized using various techniques. Flow cytometry analysis requires 3D model dissociation using enzymatic or mechanical methods.

| **Supplementary table 1. Polymers and hydrogels used in bioprinting** | | | | | |
| --- | --- | --- | --- | --- | --- |
| **Name** | **Origin** | **Crosslinking agent** | **Bioprinting**  **method** | **Role** | **Ref.** |
| **Natural or natural-derived polymers** | | | | | |
| Agarose | Red algae | Thermal | EBB | Good mechanical properties  Good stability and integrality of printed construct | [1] |
| Alginate | Brown algae or bacteria | Ionic (bivalent ions, often Ca^2+^) | EBB | Good extrusion and bioprinting abilities  (shear-thinning properties)  Good stability and integrity of printed construct | [2–7] |
| Cellulose | Plants or bacteria | - | Inkjet-based  EBB | Good mechanical properties | [8–10] |
| Chitosan | Deacetylate chitin from crustacea or fungi | - | EBB | Improve the viscosity and mechanical properties | [10,11] |
| Collagen type I | Rat tail tendons | - | EBB | Presents native cell adhesion ligands  Facilitates proliferation  Supports migration | [1,6,12–14] |
| decellularized ECM | Different tissue | - | EBB  DLP | Comprises tissue-speciﬁc biochemical attributes like cytokines and growth factors  Presents native cell adhesion ligands | [14–19] |
| Fibrinogen | Plasma | Enzymatic (thrombin) | EBB  Inkjet-based | Presents native cell adhesion ligands  Promotes proliferation, and matrix synthesis | [20–22] |
| Gelatin | Hydrolysis of  collagen | Thermal or  enzymatic (transglutaminase) | EBB | Provides mechanical stability during the printing process  Presents native cell adhesion ligands  High water-adsorbing ability  Can be used as sacrificial material and increase porosity | [3,5–7,16,23] |
| Hyaluronic acid = hyaluronan | Diverse origin (e.g. rooster comb or bacteria) | - | EBB | Good mechanical properties  Promotes proliferation and angiogenesis  Interacts with numerous cell surface markers including CD44 and RHAMM (CD168) | [24,25] |
| Matrigel^TM^ (basement membrane preparation) | Engelbreth-Holm-Swarm mouse sarcoma cells | Thermal | EBB (with temperature-controlled system) | Presents native cell adhesion ligands, growth factors  Promotes differentiation | [6,26,27] |
| Silk fibroin | Larvae | Self-gelling | EBB | Good mechanical properties  Presents native cell adhesion ligands (RGD motifs) | [9,16,28] |
| **Semi-synthetic polymers** | | | | | |
| Carboxymethylcellulose | Substitution of cellulose hydroxyl groups with carboxymethyl groups | Thermal | EBB | Good extrusion abilities | [29] |
| ColMA | Methacrylation of collagen type I | Photo-crosslinking | EBB | Allows rapid crosslinking when exposed to UV irradiation  Presents native cell adhesion ligands | [30] |
| GelMA | Methacrylation of gelatin | Photo-crosslinking | EBB  Dot extrusion  DLP  SLA | Allows rapid crosslinking when exposed to UV irradiation  Provides mechanical stability during the printing process  Presents native cell adhesion ligands  Allows cell migration | [18,24,31–34] |
| HAMA | Methacrylation of hyaluronic acid | Photo-crosslinking | EBB | Allows rapid crosslinking when exposed to UV irradiation  Good mechanical properties | [24,30,35] |
| DEAE cellulose | Etherification between cellulose hydroxyl groups and a diethylaminoethyl derivative | - | EBB | Elevate the structural stability | [36] |
| Methylcellulose | Partial substitution of cellulose hydroxyl groups with methoxy groups | Thermal | EBB (with temperature-controlled system) | Good extrusion abilities | [25,32] |
| Hydroxyethyl cellulose | Substitution of cellulose hydroxyl groups with hydroxyethyl groups | - | EBB | Good extrusion abilities  Can be functionalized | [10,37] |
| **Synthetic polymers** | | | | | |
| Carbopol (Carbomer) | Polymerization of acrylic acid | - | - | Supporting bath material  Exhibits proper shear thinning property | [38] |
| Pluronic F-127 = Poloxamer 407 | Triblock copolymer of polyethylene glycol and polypropylene oxide (PEG-PPO-PEG) | Thermal | EBB (with temperature-controlled system) | Sacrificial material | [39,40] |
| Polyethylene glycol (PEG) = polyethylene oxide (PEO) | Ethylene glycol monomers derived from petrochemical resources | Ionic, physical, or covalent agents | EBB | Can be engineered to present different adhesive ligands  Support cell viability | [41] |
| Poly (ethylene glycol) diacrylate (PEG-DA) | Modified PEG with diacrylate groups | Photo-crosslinking | EBB  SLA  DLP | Carrier scaffold | [34,42–44] |
| Polycaprolactone (PCL) | Petrochemical monomer caprolactone | - | FDM | Carrier scaffold | [20,41,45] |
| Polylactic acid (PLA) | Lactic acid from corn starch, sugarcane or beetroot | - | FDM | Carrier scaffold | [13,46–48] |

Abbreviations: ColMA: methacrylated collagen; dECM: decellularized extracellular matrix; DEAE: Diethylaminoethyl; DLP: digital light processing; EBB: extrusion-based bioprinting; FDM: fused deposition modeling; GelMA: gelatin methacrylate; HAMA: hyaluronic acid methacrylate; SLA: stereolithography.

**Supplementary figure 2. Overview of parameters to consider for the preparation of a bioink.**

**Supplementary figure 3. Schematic representation of the tumor microenvironment.**

Tumors have been recognized as well-organized ecosystems, involving constant, dynamic, and reciprocal interactions between cancer cells and the tumor microenvironment.

CAF: cancer-associated fibroblast; CAA: cancer-associated adipocytes; TAM: tumor-associated macrophages; ECM: extracellular matrix.

**REFERENCES**

1. López De Andrés J, Ruiz-Toranzo M, Antich C, Chocarro-Wrona C, López-Ruíz E, Jiménez G, et al. Biofabrication of a tri-layered 3D-bioprinted CSC-based malignant melanoma model for personalized cancer treatment. Biofabrication. 2023;15:035016. https://doi.org/10.1088/1758-5090/ac8dc6

2. Grolman JM, Zhang D, Smith AM, Moore JS, Kilian KA. Rapid 3D Extrusion of Synthetic Tumor Microenvironments. Adv Mater Deerfield Beach Fla. 2015;27:5512–7. https://doi.org/10.1002/adma.201501729

3. Chaji S, Al-Saleh J, Gomillion CT. Bioprinted Three-Dimensional Cell-Laden Hydrogels to Evaluate Adipocyte-Breast Cancer Cell Interactions. Gels Basel Switz. 2020;6:10. https://doi.org/10.3390/gels6010010

4. Lee G, Kim SJ, Park J-K. Bioprinted Multi-Composition Array Mimicking Tumor Microenvironments to Evaluate Drug Efficacy with Multivariable Analysis. Adv Healthc Mater. 2024;13:2303716. https://doi.org/10.1002/adhm.202303716

5. Kim D, Jo S, Lee D, Kim S-M, Seok JM, Yeo SJ, et al. NK cells encapsulated in micro/macropore-forming hydrogels via 3D bioprinting for tumor immunotherapy. Biomater Res. American Association for the Advancement of Science; 2023;27:60. https://doi.org/10.1186/s40824-023-00403-9

6. Swaminathan S, Hamid Q, Sun W, Clyne AM. Bioprinting of 3D breast epithelial spheroids for human cancer models. Biofabrication. 2019;11:025003. https://doi.org/10.1088/1758-5090/aafc49

7. Mondal A, Gebeyehu A, Miranda M, Bahadur D, Patel N, Ramakrishnan S, et al. Characterization and printability of Sodium alginate -Gelatin hydrogel for bioprinting NSCLC co-culture. Sci Rep. 2019;9:19914. https://doi.org/10.1038/s41598-019-55034-9

8. Markstedt K, Mantas A, Tournier I, Martínez Ávila H, Hägg D, Gatenholm P. 3D Bioprinting Human Chondrocytes with Nanocellulose–Alginate Bioink for Cartilage Tissue Engineering Applications. Biomacromolecules. American Chemical Society; 2015;16:1489–96. https://doi.org/10.1021/acs.biomac.5b00188

9. Huang L, Du X, Fan S, Yang G, Shao H, Li D, et al. Bacterial cellulose nanofibers promote stress and fidelity of 3D-printed silk based hydrogel scaffold with hierarchical pores. Carbohydr Polym. 2019;221:146–56. https://doi.org/10.1016/j.carbpol.2019.05.080

10. Maturavongsadit P, Narayanan LK, Chansoria P, Shirwaiker R, Benhabbour SR. Cell-Laden Nanocellulose/Chitosan-Based Bioinks for 3D Bioprinting and Enhanced Osteogenic Cell Differentiation. ACS Appl Bio Mater. American Chemical Society; 2021;4:2342–53. https://doi.org/10.1021/acsabm.0c01108

11. Liu Q, Li Q, Xu S, Zheng Q, Cao X. Preparation and Properties of 3D Printed Alginate^−^Chitosan Polyion Complex Hydrogels for Tissue Engineering. Polymers. 2018;10:664. https://doi.org/10.3390/polym10060664

12. Wu Z, Su X, Xu Y, Kong B, Sun W, Mi S. Bioprinting three-dimensional cell-laden tissue constructs with controllable degradation. Sci Rep. 2016;6:24474. https://doi.org/10.1038/srep24474

13. Dey M, Kim MH, Dogan M, Nagamine M, Kozhaya L, Celik N, et al. Chemotherapeutics and CAR-T Cell-Based Immunotherapeutics Screening on a 3D Bioprinted Vascularized Breast Tumor Model. Adv Funct Mater. 2022;32:2203966. https://doi.org/10.1002/adfm.202203966

14. Blanco-Fernandez B, Rey-Vinolas S, Bağcı G, Rubi-Sans G, Otero J, Navajas D, et al. Bioprinting Decellularized Breast Tissue for the Development of Three-Dimensional Breast Cancer Models. ACS Appl Mater Interfaces. 2022;14:29467–82. https://doi.org/10.1021/acsami.2c00920

15. González-Callejo P, García-Astrain C, Herrero-Ruiz A, Henriksen-Lacey M, Seras-Franzoso J, Abasolo I, et al. 3D Bioprinted Tumor-Stroma Models of Triple-Negative Breast Cancer Stem Cells for Preclinical Targeted Therapy Evaluation. ACS Appl Mater Interfaces. American Chemical Society; 2024;16:27151–63. https://doi.org/10.1021/acsami.4c04135

16. Janani G, Priya S, Dey S, Mandal BB. Mimicking Native Liver Lobule Microarchitecture In Vitro with Parenchymal and Non-parenchymal Cells Using 3D Bioprinting for Drug Toxicity and Drug Screening Applications. ACS Appl Mater Interfaces. American Chemical Society; 2022;14:10167–86. https://doi.org/10.1021/acsami.2c00312

17. Shukla P, Bera AK, Yeleswarapu S, Pati F. High Throughput Bioprinting Using Decellularized Adipose Tissue-Based Hydrogels for 3D Breast Cancer Modeling. Macromol Biosci. 2024;24:e2400035. https://doi.org/10.1002/mabi.202400035

18. Ma X, Yu C, Wang P, Xu W, Wan X, Lai CSE, et al. Rapid 3D bioprinting of decellularized extracellular matrix with regionally varied mechanical properties and biomimetic microarchitecture. Biomaterials. 2018;185:310–21. https://doi.org/10.1016/j.biomaterials.2018.09.026

19. Chen Y, Xue Y, Yan C, Jin J, Liu Y, Li J, et al. Bioprinted Fibroblast Mediated Heterogeneous Tumor Microenvironment for Studying Tumor-Stroma Interaction and Drug Screening. Adv Healthc Mater. 2025;14:e2404642. https://doi.org/10.1002/adhm.202404642

20. Piard C, Baker H, Kamalitdinov T, Fisher J. Bioprinted osteon-like scaffolds enhance in vivo neovascularization. Biofabrication. 2019;11:025013. https://doi.org/10.1088/1758-5090/ab078a

21. Cui X, Boland T. Human microvasculature fabrication using thermal inkjet printing technology. Biomaterials. 2009;30:6221–7. https://doi.org/10.1016/j.biomaterials.2009.07.056

22. Wang X, Li X, Dai X, Zhang X, Zhang J, Xu T, et al. Bioprinting of glioma stem cells improves their endotheliogenic potential. Colloids Surf B Biointerfaces. 2018;171:629–37. https://doi.org/10.1016/j.colsurfb.2018.08.006

23. Chen Q, Tian X, Fan J, Tong H, Ao Q, Wang X. An Interpenetrating Alginate/Gelatin Network for Three-Dimensional (3D) Cell Cultures and Organ Bioprinting. Mol Basel Switz. 2020;25:756. https://doi.org/10.3390/molecules25030756

24. Wang Y, Shi W, Kuss M, Mirza S, Qi D, Krasnoslobodtsev A, et al. 3D Bioprinting of Breast Cancer Models for Drug Resistance Study. ACS Biomater Sci Eng. 2018;4:4401–11. https://doi.org/10.1021/acsbiomaterials.8b01277

25. Law N, Doney B, Glover H, Qin Y, Aman ZM, Sercombe TB, et al. Characterisation of hyaluronic acid methylcellulose hydrogels for 3D bioprinting. J Mech Behav Biomed Mater. 2018;77:389–99. https://doi.org/10.1016/j.jmbbm.2017.09.031

26. Mao S, He J, Zhao Y, Liu T, Xie F, Yang H, et al. Bioprinting of patient-derived in vitro intrahepatic cholangiocarcinoma tumor model: establishment, evaluation and anti-cancer drug testing. Biofabrication. IOP Publishing; 2020;12:045014. https://doi.org/10.1088/1758-5090/aba0c3

27. Flores-Torres S, Peza-Chavez O, Kuasne H, Munguia-Lopez JG, Kort-Mascort J, Ferri L, et al. Alginate–gelatin–Matrigel hydrogels enable the development and multigenerational passaging of patient-derived 3D bioprinted cancer spheroid models. Biofabrication. 2021;13:025001. https://doi.org/10.1088/1758-5090/abdb87

28. Bandyopadhyay A, Mandal BB. A three-dimensional printed silk-based biomimetic tri-layered meniscus for potential patient-specific implantation. Biofabrication. 2019;12:015003. https://doi.org/10.1088/1758-5090/ab40fa

29. Lee J, Lee S, Lim JW, Byun I, Jang K-J, Kim J-W, et al. Development of Plum Seed-Derived Carboxymethylcellulose Bioink for 3D Bioprinting. Polymers. 2023;15:4473. https://doi.org/10.3390/polym15234473

30. Desigaux T, Comperat L, Dusserre N, Stachowicz M-L, Lea M, Dupuy J-W, et al. 3D bioprinted breast cancer model reveals stroma-mediated modulation of extracellular matrix and radiosensitivity. Bioact Mater. 2024;42:316–27. https://doi.org/10.1016/j.bioactmat.2024.08.037

31. Wei X, Huang B, Chen K, Fan Z, Wang L, Xu M. Dot extrusion bioprinting of spatially controlled heterogenous tumor models. Mater Des. 2022;223:111152. https://doi.org/10.1016/j.matdes.2022.111152

32. Rastin H, Ormsby RT, Atkins GJ, Losic D. 3D Bioprinting of Methylcellulose/Gelatin-Methacryloyl (MC/GelMA) Bioink with High Shape Integrity. ACS Appl Bio Mater. 2020;3:1815–26. https://doi.org/10.1021/acsabm.0c00169

33. Grunewald L, Lam T, Andersch L, Klaus A, Schwiebert S, Winkler A, et al. A Reproducible Bioprinted 3D Tumor Model Serves as a Preselection Tool for CAR T Cell Therapy Optimization. Front Immunol. 2021;12:689697. https://doi.org/10.3389/fimmu.2021.689697

34. Herreros-Pomares A, Zhou X, Calabuig-Fariñas S, Lee S-J, Torres S, Esworthy T, et al. 3D printing novel in vitro cancer cell culture model systems for lung cancer stem cell study. Mater Sci Eng C. 2021;122:111914. https://doi.org/10.1016/j.msec.2021.111914

35. González-Callejo P, Vázquez-Aristizabal P, García-Astrain C, Jimenez de Aberasturi D, Henriksen-Lacey M, Izeta A, et al. 3D bioprinted breast tumor-stroma models for pre-clinical drug testing. Mater Today Bio. 2023;23:100826. https://doi.org/10.1016/j.mtbio.2023.100826

36. Geevarghese R, Somasekharan LT, Bhatt A, Kasoju N, Nair RP. Development and evaluation of a multicomponent bioink consisting of alginate, gelatin, diethylaminoethyl cellulose and collagen peptide for 3D bioprinting of tissue construct for drug screening application. Int J Biol Macromol. 2022;207:278–88. https://doi.org/10.1016/j.ijbiomac.2022.02.191

37. Gospodinova A, Nankov V, Tomov S, Redzheb M, Petrov PD. Extrusion bioprinting of hydroxyethylcellulose-based bioink for cervical tumor model. Carbohydr Polym. 2021;260:117793. https://doi.org/10.1016/j.carbpol.2021.117793

38. Ning L, Shim J, Tomov ML, Liu R, Mehta R, Mingee A, et al. A 3D Bioprinted in vitro Model of Neuroblastoma Recapitulates Dynamic Tumor-Endothelial Cell Interactions Contributing to Solid Tumor Aggressive Behavior. Adv Sci Weinh Baden-Wurtt Ger. 2022;9:e2200244. https://doi.org/10.1002/advs.202200244

39. Neufeld L, Yeini E, Reisman N, Shtilerman Y, Ben-Shushan D, Pozzi S, et al. Microengineered perfusable 3D-bioprinted glioblastoma model for in vivo mimicry of tumor microenvironment. Sci Adv. 2021;7:eabi9119. https://doi.org/10.1126/sciadv.abi9119

40. Maggiotto F, Bova L, Micheli S, Pozzer C, Fusco P, Sgarbossa P, et al. 3D bioprinting for the production of a perfusable vascularized model of a cancer niche. Front Bioeng Biotechnol. 2025;13:1484738. https://doi.org/10.3389/fbioe.2025.1484738

41. Lee W, Xu C, Fu H, Ploch M, D’Souza S, Lustig S, et al. 3D Bioprinting Highly Elastic PEG-PCL-DA Hydrogel for Soft Tissue Fabrication and Biomechanical Stimulation. Adv Funct Mater. 2024;34:2313942. https://doi.org/10.1002/adfm.202313942

42. Wu D, Wang Z, Li J, Song Y, Perez MEM, Wang Z, et al. A 3D-Bioprinted Multiple Myeloma Model. Adv Healthc Mater. 2022;11:e2100884. https://doi.org/10.1002/adhm.202100884

43. Hockaday LA, Kang KH, Colangelo NW, Cheung PYC, Duan B, Malone E, et al. Rapid 3D printing of anatomically accurate and mechanically heterogeneous aortic valve hydrogel scaffolds. Biofabrication. 2012;4:035005. https://doi.org/10.1088/1758-5082/4/3/035005

44. Huang TQ, Qu X, Liu J, Chen S. 3D printing of biomimetic microstructures for cancer cell migration. Biomed Microdevices. 2014;16:127–32. https://doi.org/10.1007/s10544-013-9812-6

45. Hauptstein J, Böck T, Bartolf-Kopp M, Forster L, Stahlhut P, Nadernezhad A, et al. Hyaluronic Acid-Based Bioink Composition Enabling 3D Bioprinting and Improving Quality of Deposited Cartilaginous Extracellular Matrix. Adv Healthc Mater. 2020;9:e2000737. https://doi.org/10.1002/adhm.202000737

46. Hong S, Song JM. 3D bioprinted drug-resistant breast cancer spheroids for quantitative *in situ* evaluation of drug resistance. Acta Biomater. 2022;138:228–39. https://doi.org/10.1016/j.actbio.2021.10.031

47. Dey M, Kim MH, Nagamine M, Karhan E, Kozhaya L, Dogan M, et al. Biofabrication of 3D breast cancer models for dissecting the cytotoxic response of human T cells expressing engineered MAIT cell receptors. Biofabrication. 2022;14. https://doi.org/10.1088/1758-5090/ac925a

48. Yildirim S, Nagamine MT, Kim MH, Blanque Becceneri AB, Celik NT, Namlı İ, et al. THERAPEUTIC POTENTIAL OF TAS-115 IN 3D BREAST CANCER MODELS. Biofabrication [Internet]. 2025 [cited 2025 Nov 30]; https://doi.org/10.1088/1758-5090/ae2400
